# Enhancing Anticipatory Postural Adjustments: A Novel Approach to Balance Rehabilitation

**DOI:** 10.4172/2165-7025.1000e144

**Published:** 2016-04-29

**Authors:** Alexander S. Aruin

**Affiliations:** Department of Physical Therapy, University of Illinois at Chicago, IL, USA

**Keywords:** Balance, Rehabilitation, Anticipatory postural adjustments, Training

## Abstract

Balance impairment is common in individuals with neurological disorders and older adults and is a major cause of falls in these populations. Evidence on the effectiveness of conventional interventions for balance restoration is limited. We describe a novel approach to balance rehabilitation that is based on enhancing anticipatory postural adjustments.

## Editorial

Human vertical posture is inherently unstable because of the high location of the center of mass, small support area, and multiple joints between the feet and the center of mass. When a standing person performs a quick movement and/or interacts with external objects, the mechanical coupling of body segments leads to postural perturbations that may be destructive for fragile balance. The central nervous system (CNS) uses two main postural strategies to maintain and restore balance when a human body is perturbed. Anticipatory postural adjustments (APAs) control the position of the center of mass (COM) of the body by activating the trunk and leg muscles prior to a forthcoming body perturbation, thus minimizing the danger of losing equilibrium (reviewed in [1]). Compensatory postural adjustments (CPAs) are initiated by the sensory feedback signals and serve as a mechanism of restoration of the position of the COM after a perturbation has already occurred [2–4].

Postural control in humans is based on the effective use of anticipatory and compensatory postural mechanisms. In the case of an unexpected perturbation to posture (such as being hit in a crowded space), CPAs are the only mechanism used by the CNS to restore balance. On the other hand, when the perturbation is predictable, APAs act as the first line of defense preparing the body for the upcoming disturbance and are thereafter followed by CPAs that help in completing the process of balance restoration. As such, the utilization of APAs considerably reduces the need for large CPAs and results in greater postural stability as demonstrated by significantly smaller displacements of the body’s COM and center of pressure (COP) following a perturbation in healthy young adults [5,6]. These findings highlight the importance of APAs in control of posture and point out the existence of a relationship between anticipatory and compensatory components of postural control.

Anticipatory postural adjustments are impaired in individuals with neurological disorders such as Parkinson’s Disease [7–9], stroke [10,11], cerebral palsy [12], multiple sclerosis [13–15], in individuals with low back pain [16], lower leg amputation [17], and in older adults [18]. It was reported in the literature that an impaired APA generation is associated with larger compensatory muscle responses. Thus, diminished APAs seen in older adults require them to rely primarily on CPAs when restoring balance [18,41]. Because of the impaired APAs, older adults are more unstable than young adults and are at a higher risk of losing balance when exposed to similar perturbations [18–20]. In addition, an inability to produce APAs relates to an increased likelihood of falls in older populations, whereas older adults utilizing APAs show no difference in stability as compared to young adults [21]. Moreover, individuals with poorly coordinated or inefficient APAs show postural instability during self-initiated movements [22] and inefficient APAs during obstacle negotiation are reported to be the causes of accidental falls [23]. Furthermore, older adults with fear of falling have altered APAs [24].

While restoring APAs in people with balance deficit seems like an obvious treatment, in clinical practice, a large percentage of individuals requiring balance rehabilitation are treated with conventional rehabilitation approaches concentrated on training compensatory recovery strategies for improving postural stability [20,25]. However, new rehabilitation approaches based on retraining the ability to generate and utilize APAs could be beneficial for improving balance, mobility, independence, and quality of life of people with balance deficit.

It has been widely established that for training to be effective, the protocol needs to comply with the principle of training specificity [26]. Therefore, in order to improve the generation and utilization of anticipatory postural adjustments for enhancement of balance and mobility, it is pertinent that the training involves APA-specific activities. Previous studies demonstrating that APAs depend on the magnitude and direction of the perturbation [27–29], characteristics of voluntary action associated with a perturbation [30,31], body stability [32–35], predictability of the forthcoming perturbation [27], and fear of falling [36] provided a background for the selection of two activities that could be used for retraining APAs.

The task of ball throwing involves an internal, self-initiated perturbation and ball catching produces an externally-induced perturbation. Both tasks necessitate the generation and utilization of APAs in preparation for the upcoming disturbance (caused by the ball) in order to maintain postural stability after the disturbance. Moreover, perturbations induced by ball catch or throw are predictable (known) by the subjects as only predictable perturbations generate APAs [5,6]. Additionally, these two tasks generate perturbations at the shoulder level, mimicking common daily experiences such as being pushed on the shoulder in a crowded street or opening revolving doors.

The outcome of recent studies in young and older adults demonstrated that a single training session involving a functional activity such as catching or throwing a ball improves the generation of APAs prior to a predictable external perturbation [37,38]. Moreover, it was shown that about 120 repetitions of throwing a medicine ball was enough to see improvements in APAs generated in relation to a different task that was not used during training [37]. Furthermore, a four-week APA-based training program involving ball catching activities resulted in improved clinical outcome measures of balance in older adults participating in APA-based activities as compared to control subjects who did not receive APA-focused training [39,40].

Substantial evidence suggests that a decreased ability to generate and utilize anticipatory postural adjustments is linked to balance impairment and that anticipatory postural adjustments could be enhanced with training. While additional studies are needed to define the specifics of the training protocols, there is no doubt that APA-based interventions can be an effective rehabilitation approach in improving postural control, functional balance, mobility, and quality of life in individuals with balance deficit.
